# New Biomarkers and Their Potential Role in Heart Failure Treatment Optimisation—An African Perspective

**DOI:** 10.3390/jcdd9100335

**Published:** 2022-10-02

**Authors:** Marheb Badianyama, Dineo Mpanya, Umar Adamu, Farai Sigauke, Samantha Nel, Nqoba Tsabedze

**Affiliations:** Division of Cardiology, Department of Internal Medicine, School of Clinical Medicine, Faculty of Health Sciences, University of the Witwatersrand, Johannesburg 2193, South Africa

**Keywords:** heart failure, biomarker, protein biomarker, natriuretic peptides, cardiac troponin, prognosis, non-coding RNA, genetic risk score

## Abstract

Heart failure is a clinical syndrome resulting from various cardiovascular diseases of different aetiologies and pathophysiology. These varying pathologies involve several complex mechanisms that lead to the activation of the neurohumoral system, inflammation, angiogenesis, apoptosis, fibrosis, and eventually adverse cardiac remodelling associated with a progressive decline in cardiac function. Once a diagnosis is made, the cardiac function has a gradual decline characterised by multiple hospital admissions. It is therefore imperative to identify patients at different stages of the heart failure continuum to better risk stratify and initiate optimal management strategies. Biomarkers may play a role in the diagnosis, prognostication, and monitoring response to treatment. This review discusses the epidemiology of heart failure and biomarkers commonly used in clinical practice such as natriuretic peptides and cardiac troponins. In addition, we provide a brief overview of novel biomarkers and genetic coding and non-coding biomarkers used in the management of patients with heart failure. We also discuss barriers that hinder the clinical application of novel biomarkers. Finally, we appraise the value of polygenic risk scoring, focusing on sub-Saharan Africa.

## 1. Introduction

Heart failure (HF) is a complex clinical syndrome resulting from various cardiovascular diseases of different aetiologies [1]. The underlying pathophysiological mechanisms activate the neurohumoral system, inflammation, angiogenesis, apoptosis and fibrosis [2]. In addition, the cardiac myocyte undergoes adverse cardiac remodelling, associated with a progressive decline in cardiac function [3].

The clinical diagnosis of heart failure may be challenging, with some patients presenting with nonspecific symptoms resulting in delays in accurate diagnosis. Once a diagnosis is made, cardiac performance steadily declines and is characterized by multiple rehospitalisations secondary to acute on chronic decompensation. Despite significant advances in therapy, the 5-year all-cause mortality rate is approximately 50% [4]. Early diagnosis and timely initiation of guideline-directed medical therapy (GDMT) are paramount to mitigating these poor outcomes. Heart failure biomarkers may play a significant role in diagnosis, phenotyping, risk stratification, prognostication, and monitoring of the response to therapy [5,6].

The American National Institute of Health Bethesda Biomarkers Definitions Working Group defines a biomarker as “a characteristic that is objectively measured and evaluated as an indicator of normal biological processes, pathogenic processes, or pharmacologic responses to a therapeutic intervention” [7]. Novel biomarkers may provide a low-cost, non-invasive, measurable point of care to confirm or exclude a HF diagnosis [8]. This narrative review will discuss the use of HF biomarkers in the diagnosis, risk stratification, prognostication, and therapeutic monitoring of cardiac failure in Sub-Saharan Africa (SSA). 

## 2. Epidemiology of Heart Failure

Heart failure is associated with higher recurrent hospitalisations and mortality rates in sub-Saharan Africa (SSA) [9]. In a systematic review and meta-analysis comprising 35 studies on HF in SSA, the most common causes of HF affecting a young population in the third and fifth decades of life were hypertensive heart disease, affecting 39.2% [95% confidence interval (CI) = 32.6–45.9]), followed by cardiomyopathies (21.4% [95% CI = 16.0–27.2]), rheumatic heart disease (14.1% [95% CI = 10.0–18.8]) and ischemic heart disease (7.2% [95% CI = 4.1–11.0]) [10]. 

In addition to these causes, human immunodeficiency virus (HIV)-associated cardiovascular disease and tuberculous pericarditis have been reported as other important causes of HF in this region [11]. This contrasts with reports from high-income countries (HIC), where ischaemic heart disease is the most common cause of HF, affecting an older population in the seventh decade of life [12,13]. 

Although there have been no population-based epidemiological studies or studies reporting on the incidence of heart failure in Africa, data from hospital-based studies provide meaningful insights into its prevalence [9,10,14]. The Heart of Soweto cohort study assessed the burden and clinical features of heart failure in over 4100 participants and demonstrated that females were more likely to be affected (59%) than men. Also, females diagnosed with heart failure were slightly younger than their male counterparts, with a mean age of 53 vs. 55 years, respectively [15]. Published data from studies in high-income countries have reported that decompensated heart failure carries a one-year all-cause mortality rate of 23% and a five-year all-cause mortality rate of 50% [16]. In SSA, the in-hospital mortality rate of decompensated HF is up to 8.3% which is similar to reports from HIC [9,10]. 

## 3. Biomarkers

This section will appraise traditional and novel biomarkers for HF diagnosis, risk stratification, and prognosis. The B-type natriuretic peptide (BNP), N-terminal pro B-type natriuretic peptide (NT-proBNP), and cardiac troponins will be discussed in more detail based on the robust clinical evidence available. Finally, we will focus on their potential ability to guide and thereby optimise HF treatment in African populations.

The 2022 American College of Cardiology, American Heart Association and the Heart Failure Society of America (ACC/AHA/HFSA) and the 2021 European Society of Cardiology (ESC) HF guidelines recommend using natriuretic peptides (NP) to support the diagnosis of HF [1,17]. Due to their high negative predictive value between 0.94 and 0.98 [18,19,20], the ESC recommends measuring NP to rule out acute HF at precise thresholds (BNP < 100 pg/mL, NT-proBNP < 300 pg/mL or mid-regional pro-atrial natriuretic peptide (MR-proANP) < 120 pmol/L), and chronic HF (BNP < 35 pg/mL, NT-proBNP < 125 pg/mL or MR-proANP < 40 pmol/L) [1]. Importantly, these thresholds exclude HF rather than confirm the diagnosis of HF. 

The current ACC/AHA/HFSA HF guidelines give a class I recommendation for using BNP or NT-proBNP to prognosticate HF once diagnosed. However, the ESC guideline does not recommend measuring BNP or NT-proBNP for prognostication but acknowledges their potential usefulness [1]. In addition, the ACC/AHA/HFSA recommends using NP exclusively [17]. On the other hand, the 2021 ESC guidelines for the diagnosis and treatment of acute and chronic heart failure recommend measuring cardiac troponins for the diagnosis of acute HF to exclude acute coronary syndrome (ACS) as an underlying cause of HF [1]. Table 1 summarises the biomarkers proposed by these guidelines and their clinical uses in HF.

We performed a systematic literature search of studies investigating the use of biomarkers in heart failure patients residing in Africa. We searched PubMed, Scopus and Web of Science for original research articles published to date using the search terms “heart failure” AND “biomarker” AND “Africa.” The literature search identified 141 studies. After screening full-text articles, six studies were included and are reported in Table 2. In addition, the Preferred Reporting Items for Systematic Reviews and Meta-Analyses (PRISMA) 2020 flow diagram is attached as a Supplementary File (Figure S1).

### 3.1. BNP Monitoring in Heart Failure

B-type natriuretic peptide (BNP) and N-terminal pro-B-type natriuretic peptide (NT-proBNP) are the gold standard biomarkers used for the diagnosis of HF [27]. In addition, BNP and NT-proBNP are the only biomarkers to date which provide additional value to the standard clinical laboratory data of HF [28]. However, their ability to guide pharmacotherapy through the titration of HF medications based on BNP or NT-proBNP plasma levels is limited.

In SSA, Sani and colleagues included a subset of 80 African patients with acute HF enrolled in the Bi-treatment with hydralazine/nitrates vs. placebo in Africans admitted with acute HF (BAHEF) trial [23]. The study showed that baseline NT-proBNP predicts combined cardiovascular mortality or HF hospitalisation at six months (hazard ratio [HR]: 2.12; 95% CI 1.06–4.22; *p* = 0.0328). The study demonstrated that a change in NT-proBNP levels over time is associated with an improved NYHA functional class at six months and could thus stratify African patients with acute HF. However, the study sample size was small, with a short follow-up period. Furthermore, how changes in this biomarker could guide the titration of pharmacotherapy in these patients remained largely undefined. 

Similarly, a recent prospective cohort of 35 South African women with acute peripartum cardiomyopathy (PPCM) found that raised NT-proBNP ≥ 900 pg/mL at baseline strongly predicts failure to recover the LVEF (odds ratio [OR]: 0.20, 95% CI 0.04–0.89; *p* = 0.035) at one year [25]. However, while confirming the prognostic utility of this biomarker, the study did not assess its role as a marker of HF therapeutic response in African subjects diagnosed with HF.

The Guiding Evidence Based Therapy Using Biomarker Intensified Treatment in Heart Failure (GUIDE-IT) multi-centre randomized clinical trial, which included 894 adults from HIC with HFrEF, concluded that NT-proBNP guided treatment does not offer superior benefit compared to the standard care of HFrEF [29]. In this study, treatment guided by NT-proBNP plasma levels did not improve the time-to-first HF hospitalisation nor the cardiovascular death rate (adjusted hazard ratio [HR], 0.98; 95% CI 0.79–1.22; *p* = 0.88). 

However, the GUIDE-IT study recruited patients with high-risk characteristics. For example, patients had an average baseline NT-proBNP level of 2607 pg/mL and a history of at least one HF event within the prior year. Therefore, participants were likely to develop adverse drug events such as cardiogenic shock and renal failure when the HF medication dosage was up-titrated. These high-risk characteristics may have restricted the optimal titration of pharmacotherapy in the NT-proBNP-guided treatment group, hence reducing the differences between the two groups. Moreover, the study was conducted in the United States and Canadian sites with substantial expertise in HF care. Participants had intense follow-up clinic visits, which may have further masked any possible differences between the treatment groups. Subsequently, the study was terminated prematurely because it failed to show significant differences between NT-proBNP guided therapy and standard HF care.

A patient-level meta-analysis of nine clinical trials, which included 2000 patients with chronic heart failure with reduced ejection fraction (HFrEF), showed that NT-proBNP-guided treatment significantly reduced all-cause mortality (hazard ratio [HR], 0.62; 95% CI 0.45–0.86; *p* = 0.009) compared to conventional HF care [30]. The study also showed that this biomarker-guided strategy significantly reduced HF hospitalisation (hazard ratio [HR], 0.80; 95% CI 0.67–0.94; *p* = 0.009), and improved survival in patients younger than 75 years (hazard ratio [HR], 0.62; 95% CI 0.45–0.85; *p* = 0.004) but not in older patients (hazard ratio [HR], 0.98; 95% CI 0.75–1.27; *p* = 0.96). Notably, the meta-analysis by Troughton et al. consisted of individual patient data sought directly from original investigators rather than aggregated data obtained from research publications. Studying individual patient data allowed the researchers to standardize the clinical outcomes definitions and mitigate patient characteristics that could have directly influenced outcomes or treatment titration. In addition, their findings had no heterogenicity despite the differences in the study designs. 

The Ahmadu Bello University B-type Natriuretic Peptide (ABU-BNP) study assessed the clinical value of monitoring changes in BNP levels and echocardiographic pulse-wave tissue doppler (TD) left ventricular filling pressure (E/”) in 75 Nigerian patients with acute HF managed for one month with standard HF care [22]. There was a significant reduction in BNP levels of 38.9% from 450 to 275.0 pg/mL (*p* < 0.001) at 30 days. Notably, the reduction in BNP levels was associated with significant improvement in TD-derived E/” of 17.7 at baseline and 11.2 at 30 days (*p* < 0.001). In other words, the study showed that this soluble biomarker could assess the effectiveness of HF drug therapy and further optimise treatment in African subjects. This sub-Saharan African study supports previous studies from HIC, which reported that a reduction in BNP or NT-proBNP levels of ≥30% from baseline signals a better prognosis and might help monitor response to HF pharmacotherapy [31,32]. 

Clinical data from the ABU-BNP study was collected from a single-centre, and they had a small number of patients studied over a short duration [22]. Therefore, we encourage validating these findings in a larger multi-centre cohort that includes African subjects followed over an adequate period. More importantly, the study excluded patients who did not improve clinically after four weeks. Hence, the study provided no comparison between the association of changes in BNP levels and clinical outcomes of African HF patients who improved compared to those who did not at 30 days. Furthermore, while the study showed that serum BNP could be measured to monitor the response to HF treatment and improvements in NYHA functional capacity, the study did not explore the relationship between BNP-guided treatment response and hard outcomes such as mortality.

Zile et al.’s study also assessed the clinical value of monitoring changes in NT-proBNP levels from baseline during the follow-up of 2080 HF patients from HIC [33]. The patients were enrolled in The Prospective Comparison of Angiotensin receptor-neprilysin inhibitor (ARNI) with Angiotensin-converting enzyme inhibitor (ACE-I) to Determine Impact on Global Mortality and Morbidity in Heart Failure (PARADIGM-HF) trial [34]. The study showed that cardiovascular morbidity and mortality corresponded with changes in plasma NT-proBNP levels over time. In other words, regardless of the treatment intervention, patients with significantly reduced NT-proBNP levels had proportionally lower cardiovascular death rates or HF-related hospitalisations. However, there are mixed results from various clinical trials which assess whether changes in NP levels from baseline over time could help determine the response to therapy [35,36,37,38,39,40]. 

**Figure 1:**
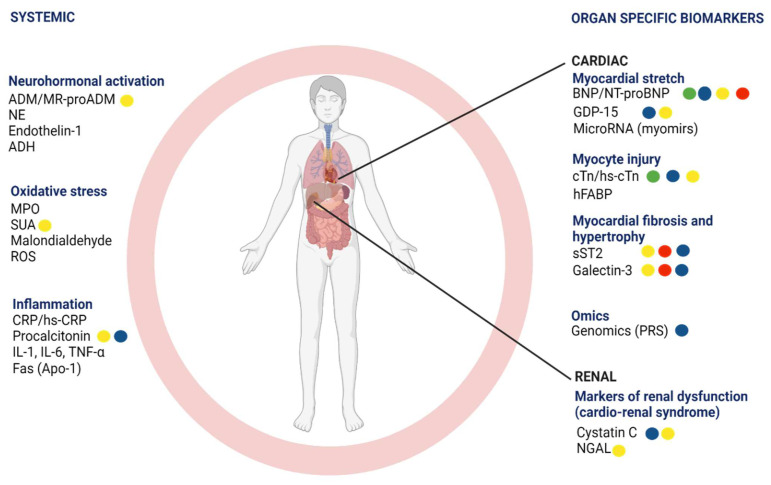
Biomarkers involved in various pathophysiological pathways of heart failure and their potential clinical uses. The green circle refers to biomarkers used for screening, the blue circle for phenotyping, the yellow circle for prognosis and risk stratification, and the red circle refers to biomarkers used for treatment monitoring. ADH—anti-diuretic hormone; ADM—adrenomedullin; BNP—B-type natriuretic peptide; CRP—C-reactive protein; cTn—cardiac troponin; Fas (Apo-1)—Fas apolipoprotein-1; GDP-15—growth differentiation factor-15; hFAB—heart-type fatty acid binding protein; hs-CRP—high-sensitivity C-reactive protein; hs-cTn—high-sensitivity cardiac troponin; IL-1—interleukin-1; IL-6—interleukin-6; MicroRNA—microscopic ribonucleic acid; MPO—myeloperoxidase; MR-proADM—mid-regional pro-adrenomedullin; NE—norepinephrine; NT-proBNP—N-terminal pro-B-type natriuretic peptide; NGAL—neutrophil gelatinase-associated lipocalin; PRS—polygenic risk scores; ROS—reactive oxygen species; sST2—soluble source of tumorigenicity 2; SUA—serum uric acid. Permission to use the figure obtained from BioRender.com.

Lastly, although BNP and NT-proBNP remain the diagnostic gold standard for HF, there is conflicting evidence surrounding their utility in treatment optimization. Therefore, research has increased to study other biomarkers involved in the pathophysiology of HF, which could potentially help manage this complex syndrome. Figure 1 illustrates the established and new biomarkers classified into eight pathophysiological pathways of HF and their potential clinical utilities in HF using a colour-coded scheme.

### 3.2. High-Sensitivity Cardiac Troponin Monitoring

High-sensitivity cardiac troponins (hs-cTn) are elevated in HF through various mechanisms, which occur irrespective of the presence or absence of myocardial ischaemia secondary to obstructive coronary artery disease [41,42]. In acute HF, transient ventricular pressure overload causes myocardial injury, increasing the hs-cTn plasma levels [43,44]. Nearly 98% of individuals with acute HF have raised hs-cTn concentrations, with approximately 81% of cases above the upper limit of normal (ULN) [45]. In addition, continuous left ventricular end-diastolic pressure overload in chronic HF results in left ventricular hypertrophy (LVH). The LVH leads to subendocardial hypoperfusion and eventually subendocardial ischaemia, resulting in increased levels of plasma hs-cTn. A large cohort of 9289 chronic HF patients reported that the average concentration of high-sensitivity cardiac troponin T (hs-cTnT) was 16 ng/L, with > 50% of patients above the upper limit of normal (ULN) [46].

The measurement of hs-cTn is required to diagnose acute HF, excluding type 1 myocardial infarction (MI) or myocardial injury [5]. A type 1 MI is most likely the precipitator of any HF episode when hs-cTn levels are more than ten times the ULN or when there is a significant increase, for example, above 100 ng/L within 1–3 h, in the presence of signs and symptoms of myocardial ischaemia [1]. In addition, elevated hs-cTn at admission predicts the risk for cardiac remodelling and cardiovascular mortality in both acute and chronic HF syndromes [45,46].

Elevated hs-cTn may help screen seemingly healthy adults for the onset of HF. A study involving 4221 subjects showed that hs-cTnT concentrations above 13 ng/L were associated with an HF incidence rate of 6.4 per 100 person-years (95% CI 5.8–7.2) and a risk for HF (adjusted hazard ratio, [HR] 2.48; 95% CI 2.04–3.00) [47]. In this study, elevated hs-cTnT predicted cardiovascular death compared to normal hs-cTnT levels (adjusted hazard ratio, [HR] 2.91; 95% CI 2.37–3.58). Repeated measurement of hs-cTnT at 2–3 years offered better risk stratification. For example, a change in hs-cTnT that was more than 50% from baseline was associated with an even higher risk of developing HF (adjusted HR = 1.61; 95% CI 1.32–1.97) and higher cardiovascular mortality (adjusted HR 1.65; 95% CI 1.35–2.03).

Similarly, a study including 236 out of 1767 heart failure with preserved ejection fraction (HFpEF) patients enrolled in the Treatment of Preserved Cardiac Function Heart Failure with an Aldosterone Antagonist Trial (TOPCAT) [48], showed that increased high-sensitivity cardiac troponin I (hs-cTnI) increases the risk of the composite endpoint of cardiovascular death and HF hospitalisation (HR 1.42; 95% CI 1.20–1.69; *p* < 0.001).

Furthermore, hs-cTnT independently predicts the risk of all-cause mortality in acute decompensated HF, irrespective of NT-proBNP and soluble source of tumorigenicity 2 (sST2) (HR 1.16; 95% CI 1.09–1.24; *p* < 0.001) [49]. Importantly, using a multi-marker strategy that includes hs-cTnT and NP provides a superior prediction of outcomes [50].

To our knowledge, no study has shown how hs-cTn informs clinical decision-making in chronic HF. Although previous studies have suggested that persistently elevated hs-cTn could guide the intensity of follow-up and the titration of pharmacotherapy, this remains speculative, with no primary studies originating from SSA. The major HF practice guidelines do not recommend measuring hs-cTn to guide or monitor HF treatment in patients with chronic HF. However, a raised hs-cTn in chronic HF syndrome may warrant a clinical reassessment, modifying the patient’s risk factors and optimizing pharmacotherapy.

### 3.3. Novel Protein, Non-Coding, Genetic and Exhaled Biomarkers

Given the proven diagnostic value of BNP and NT-proBNP, the focus on discovering new HF biomarkers has steered toward prognostication rather than diagnosis. As shown in Figure 1, several biomarkers have been studied, and many are beyond the scope of this review. Therefore, we focus on contemporary protein, non-coding, genetic, and exhaled biomarkers that could potentially improve HF prognostication and therapeutic monitoring in clinical practice.

#### 3.3.1. Soluble Source of Tumorigenicity 2

Soluble source of tumorigenicity 2 (sST2) is a new protein biomarker associated with myocardial inflammation, fibrosis, and hypertrophy in HF [51]. However, increased circulatory sST2 is not diagnostic of HF due to its low cardiac specificity and associations with other extra-cardiac conditions such as lung diseases [52]. 

Multiple studies in HIC reported that an elevated sST2 baseline concentration predicts all-cause and cardiovascular death [53,54,55]. This increased risk is particularly true for acute HF patients with New York Heart Association (NYHA) class 3 or 4, renal dysfunction and markedly elevated NT-proBNP [38,40]. Similarly, Socrates et al. reported that sST2 is an independent predictor of one-year mortality in acutely decompensated HF but not in chronic HF [56]. However, results from several clinical trials are inconclusive on whether combining sST2 and NT-proBNP improves the prediction of clinical outcomes of HF compared to using them individually.

A sizeable cross-sectional Tanzanian study enrolled 388 antiretroviral therapy (ART) -naïve human immunodeficiency virus (HIV)- infected and 461 healthy adults to determine the association between serum sST2 levels and HIV-associated myocardial diastolic dysfunction, a known precursor of HFpEF [24]. The study revealed that HIV-infected adults had a higher prevalence of diastolic dysfunction (adjusted odds ratio [OR]: 2.71, 95% CI 1.62–4.55; *p* < 0.0001), and higher sST2 levels compared to the control group (19.6 vs. 16.1 ng/mL; *p* = 0.002). These findings suggest that this biomarker may play a role in the pathogenesis of HIV-associated myocardial diastolic dysfunction prior to ART initiation. Most importantly, sST2 may be a crucial biomarker for the risk stratification, phenotyping, prognosis, and HF therapeutic monitoring of African HF patients. 

Notably, although this cross-sectional study recruited subjects who did not have a diagnosis of overt HF or meet the Framingham criteria, a small number of these subjects could have had subclinical HF. In addition, the study excluded adults with HIV-associated myocardial systolic dysfunction, a vital precursor of HFrEF. Furthermore, it is unclear whether HIV-mediated immune cell alterations or HF-specific pathways lead to the increased sST2 levels observed in HIV-infected adults with diastolic dysfunction. Nonetheless, these results are relevant in SSA, where the HIV-attributable cardiovascular risk is highest, with HF being one of the common cardiovascular manifestations of HIV [57,58]. 

Lastly, there is scant evidence for using sST2 in HF treatment monitoring. The recent sST2 As help for the management of Diagnosis, Evaluation and management of HF (STADE-HF) pilot study showed that sST2-guided therapy does not reduce HF readmissions [59]. Therefore, further research is warranted to ascertain its use in guiding clinical practice.

#### 3.3.2. Galectin-3

Galectin-3 is not cardiac-specific, and it is unclear which organs contribute to its circulatory levels and to what extent in HF [27]. The Prevention of Renal and Vascular End-Stage Disease (PREVEND) study and the Framingham Offspring Cohort showed that raised plasma levels of galectin-3 increases the risk for new-onset HF and all-cause mortality, notwithstanding other clinical factors, including NP levels [60,61]. These results were consistent with a meta-analysis that included 30,000 subjects with HF, which revealed that galectin-3 predicts the development of new-onset HF [62]. Notably, French B. et al. found that this biomarker is the strongest predictor of adverse events within five years in patients with HFpEF [63]. Data from HIC support the clinical utility of galectin-3 for HF prognostication when included in a multi-biomarker model only. However, there is presently no evidence supporting the use of galectin-3-guided therapy in HF [64,65]. 

In SSA, the study by Sani and colleagues reported that galactin-3 at baseline predicts the combined outcome of cardiovascular death or HF hospitalisation at six months, with galectin-3 being more predictive than NT-proBNP (HR, 2.81, 95% CI 1.16–4.79; *p* = 0.0001 for galectin-3, and HR 2.12, 95% CI 1.06–4.22; *p* = 0.0328 for NT-proBNP, respectively) [23].

On the contrary, Srivatsan et al. showed that elevated plasma galectin-3 levels do not predict all-cause mortality [66]. Therefore, based on its lack of specificity and modest evidence, the current 2022 ACC/AHA/HFSA HF management guideline no longer recommends measuring galectin-3 in clinical practice [17]. This lack of recommendation is a change in stance from its former class IIb recommendation for additive risk stratification in the 2017 ACC/AHA HF management guidelines [67].

#### 3.3.3. Heart-Type Fatty Acid-Binding Protein (H-FABP)

Heart-type fatty acid-binding protein (H-FABP) is also known as a mammary derived-growth inhibitor and the best-known member of the FABP family that is highly expressed in the cardiomyocytes compared to skeletal muscles and tubular cells. It is a low-molecular-weight protein involved in cellular fatty acid metabolism, cellular growth, and proliferation processes [68]. Heart-type fatty acid-binding protein is rapidly released following an injury due to its size and location in the cytoplasm. Lichtenauer et al. enrolled 65 patients with dilated cardiomyopathy and 59 patients with ischemic cardiomyopathy, and found that H-FABP levels were significantly higher in patients with HF compared to controls (*p* < 0.0001) [69]. In addition, H-FABP levels correlated with NYHA functional class and inversely with the left ventricular ejection fraction. In a recent narrative review by Rezar et al., it was suggested that H-FABP should be used in the risk evaluation of adverse cardiac events. In the future, H-FABP may be applied in the early detection of ischemia, worsening of renal failure, and long-term treatment planning [70].

#### 3.3.4. Growth Differentiation Factor-15

Growth differentiation factor-15 (GDF-15), also known as macrophage inhibitory cytokine-1 (MIC-1), is a new biomarker released following myocardial ventricular wall stretch [27].

The most promising clinical utility of plasma GDF-15 levels is monitoring response to left ventricular assist device (LVAD) implantation in advanced HF. Previous studies showed that the implantation of LVAD leads to a rapid and significant decline in the circulatory GDF-15 concentrations of patients with advanced HF [71,72]. Therefore, measuring the plasma concentration of GDF-15 post LVAD implantation could potentially help monitor therapeutic response. However, the utility of GDF-15 in guiding pharmacological treatment is unclear, and there have been no reports from SSA. 

#### 3.3.5. Glutathione Transferase (GST) P1

Glutathione S-transferase (GST) P1 is an isozyme of the glutathione S-transferase family that regulates cellular homeostasis and detoxifies metabolites such as reactive oxygen species and inflammation [73]. Glutathione S-transferase P1 is detected in high quantities in the serum of individuals with heart failure and was found to be a strong predictor of myocardial infarction mortality, CV events, and HF admission [74,75,76]. Also, GST P1 levels were significantly higher in patients with end-stage HF than in controls in a study of 193 patients subdivided based on the LVEF. Even though GST P1 and NT-proBNP are associated with NYHA functional class 3 and 4, GST P1 better diagnosed HF in patients with a LVEF ≤ 42%. Glutathione S-transferase P1 is an independent, sensitive and specific predictor of LV function in HF than NT-proBNP. The serum levels of GST P1 ≤ 126 ng/mL, identified HF patients with a LVEF ≤ 42% with 90% sensitivity and 95% specificity, while-proBNP at ≤396 pg/mL level had 97% sensitivity and 20% specificity [73]. However, more research is still needed to clarify the relationship between GST P1 and HF clinical characteristics and application in clinical practice [76].

#### 3.3.6. Circulating Ketone bodies and Heart Failure

The heart has remarkable metabolic flexibility that can produce reduction equivalents orfuel based on the demands, neurohormonal status, and the availability of substrates [77,78,79,80]. The circulating levels of ketone bodies are increased in patients with acute and chronic HF and do not affect the oxidation rates of fatty acids or glucose [77,78,80]. The relationship between ketone bodies and novel biomarkers has been consistent but varied in its use in assessing the severity of HF. In a study by Kashiwagi et al. of 1030 patients who underwent cardiac catheterization for various cardiovascular disorders, total ketone bodies positively correlated with BNP regardless of the levels and not with haemodynamic parameters [81]. The finding suggests that BNP might induce the elevation of total ketone bodies, and by extension, the modulation of circulating ketone levels may represent a novel treatment principle in patients with heart failure [81,82].

#### 3.3.7. MicroRNAs

MicroRNAs (miRNA) are small non-coding single-stranded RNA molecules, between 21–25 nucleotides in length. They regulate gene expression by inhibiting protein translation and the degradation of the messenger RNA (mRNA) [83,84]. However, in HF, these molecules are differentially regulated and contribute to several pathophysiological processes, including cardiomyocyte hypertrophy, fibrosis, alterations in calcium handling, and regression to a fetal gene programme [85]. In addition, miRNAs specific to the heart muscle, known as cardiac “myomir”, are released into the plasma in response to myocardial injury or an increased myocardial stretch [86]. 

A common finding across several previous studies is that baseline levels of cardiac miRNAs may help to distinguish patients who would be the most likely to respond successfully to LVAD therapy. In addition, Melman et al. found that miRNA-30d could be a marker of prognosis and therapeutic response to cardiac resynchronization therapy (CRT) in patients with advanced HF [87]. Notably, this study also showed that miRNA-30d was a better clinical predictor of CRT response than other clinical variables, for example, the duration of QRS on an electrocardiogram.

Similarly, Sucharov et al. identified a set of miRNAs (miRNA 208a-3p and miRNA-591) differentially expressed in HF patients who respond favourably to beta-blockers compared to those who do not [88]. These findings could potentially improve HF risk stratification and treatment optimization. Therefore, although the evidence of this new biomarker stems primarily from animal studies, the use of miRNA-guided therapy in HF has promising translational potential for HIC and SSA. 

#### 3.3.8. Novel Genetic Biomarkers

A crucial area of research in HF is the need to shift the use of biomarkers from diagnosis and prognosis towards improved selection and titration of treatment for precision medicine, which has unlocked a broad and intricate field of research known as the’’omic” approach. Omics integrate several biological disciplines from the genome (genomics) to transcriptome (transcriptomics), proteome (proteomics), metabolome (metabolomics), epigenome (epigenomics), and microbiome (microbiomics) [89,90].

Genomic approaches have been incorporated in several genome-wide association studies (GWAS) to evaluate how the human genome plays a role in the development of HF syndrome. Genome-wide association studies combine predictive coronary artery disease risk scores and single nucleotide polymorphisms (SNPs) of various genes [91]. As a result, several HF genotypes have been described. Through this approach, research has found specific genomic loci (CLCNKA, BAG3, HSPB7) associated with HF [92,93], SNPs of genes encoding enzymes associated with oxidative stress and LVEF, as well as genotypes of GNB3 related to HF onset and progression [94]. In addition, GWAS have identified numerous genetic differences between patients diagnosed with HF and those suffering from other cardiovascular diseases [68,95]. Potentially, this could help identify previously unrecognizable phenotypes of HF and inform novel therapeutic strategies.

An important gene studied in African subjects with HFpEF is the leptin (LEP) gene and leptin receptor (LEPR) gene polymorphism. El-Aziz and colleagues genotyped 100 Egyptian dyslipidaemic subjects with coronary artery disease and HFpEF compared to 100 healthy subjects (control group) for LEP and LEPR gene polymorphism [21]. The study showed that HFpEF is associated with increased serum leptin levels, and the LEP AA and LEPR RR genotypes carry at least a threefold increased risk of developing HFpEF compared to the control group (OR 3.9, 95% CI 1.2–12.6; *p* = 0.02) for LEP AA genotype and, OR 3.7, 95% CI 1.4–10.3; *p* = 0.007) for LEPR RR genotype, respectively. However, these results cannot be generalized given the study’s lack of ethnic diversity. In addition, the study did not correct the serum leptin levels measured to total or regional body fat. Thus, the validity of these findings is limited.

However, most GWAS investigate SNP-phenotype associations related to a single genetic variant. As a result, this undermines the fact that HF is affected by more than one genetic variant. Polygenic risk scores as HF biomarkers far better reflect variabilities in genetic and epigenetic characteristics of HF development and progression compared to single genetic scores used in GWAS [96,97]. This strategy promises to better risk stratify the patient’s HF-risk and individualise treatment based on the patient’s genetic makeup. 

#### 3.3.9. Exhaled Acetone

The exhaled breath is a complex molecule medium that may provide health status information [98,99,100]. There are several of these that are used in the diagnosis and the assessment of heart failure severity [99,100]. Patients with acute decompensated HF had significantly higher breath concentrations of acetone compared to the controls (β = 0.53, *p* < 0.0001) [99]. Kupari et al., compared patients with HF and normal controls, and concluded that HF might have predisposed the participants to ketone formation after an overnight fast. However, in their study, no comparison was made with other biomarkers [99]. However, the study by Marcondes-Braga et al. that included 235 HF patients investigated the use of exhaled acetone in the diagnosis of HF and as a biomarker to determine its severity. They reported that the concentration of exhaled breath acetone was higher in patients with HF compared to the controls (3.7 mg/L; IQR 1.69–10.45 mg/L) vs. 0.39 mg/L; IQR, 0.30–0.79 mg/L) and differed significantly to the severity of HF assessed by the New York Heart Association classification. In addition, the diagnostic accuracy and sensitivity of exhaled breath acetone to diagnose HF with decompensation was similar to that obtained with B-type natriuretic peptide (85%) [100]. In another follow-up study of 89 patients with HF and reduced ejection fraction for one year, these authors reported that exhaled breath acetone of ≥ 3.7μg/L was associated with a worse prognosis (*p* = 0.001). Furthermore, the exhaled breath acetone above 3.7μg/L increased the risk of death or transplantation by three-fold (HR = 3.26, 95%CI = 1.56–6.80, *p* = 0.002) [101].

### 3.4. Factors That Limit The Clinical Application of New Biomarkers

**Figure 2:**
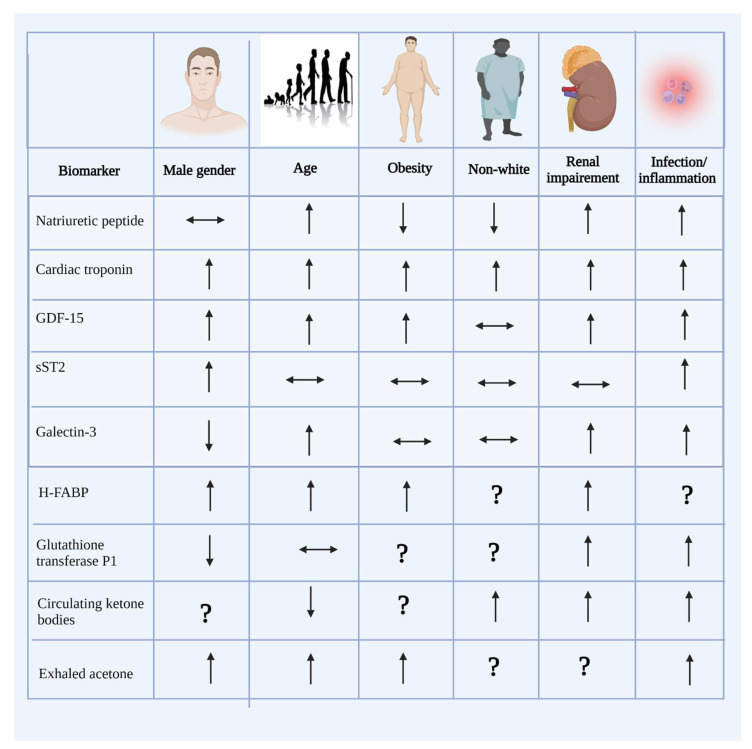
Effects of confounders on biomarker level. GDF-15—growth differentiation factor-15; sST2—soluble suppression of tumorigenicity 2. H-FAB—Heart-Type Fatty Acid-Binding Protein. Factors increasing (arrow facing up) and decreasing (arrow facing down) biomarker levels. Unchanged biomarker levels are shown by an arrow facing both ways. An unknown effect on biomarker level is shown by a question mark.

The most significant limitation to implementing new biomarkers of HF in clinical practice is their lack of cardiac specificity. Since other organs also release these new biomarkers, it is difficult to directly correlate them to cardiac function and specific indicators of HF progression. Also, biomarker levels may be influenced by other clinical factors such as male gender and obesity (Figure 2). Thus, it is challenging to inform clinical decision-making for the diagnosis, risk stratification, prognosis, and treatment of HF.

Studies reporting that new biomarkers add prognostic or therapeutic value in managing HF arise from pre-clinical mouse models. In addition, most clinical trials reporting the benefits of biomarker-guided HF therapy are single-blinded, with considerable risks of bias and conflicting results. Therefore, although promising, their implementation in clinical practice has been unsuccessful.

Multi-marker panels could improve diagnostic accuracy, prognostication, and treatment monitoring. However, this approach could delay HF diagnosis and compound healthcare costs, particularly in SSA.

## 4. Future Research and Recommendations

Given the evidence supporting multi-marker models, we propose considering this approach for the risk stratification and prognostication of HF with clinical assessment. Future research should ideally focus on developing a multi-marker strategy that is both cardiac-specific and cost-effective. However, this approach could increase the complexity of assessing an already multifaceted syndrome.

Most clinical trials showing the benefits of biomarker-guided therapies study patients with HFrEF only. Patients with HFmrEF and HFpEF are significantly under-represented. Therefore, we propose that future HF biomarker studies enrol HFmrEF and HFpEF populations. To the best of our knowledge, no sizeable primary study has been conducted on the clinical utility of HF biomarkers in treating patients from SSA. Previous African studies assessing the utility of monitoring BNP levels have included less than 100 patients. We cannot firmly extrapolate data from studies conducted predominantly among Caucasian HF patients to non-Caucasian populations. Therefore, more comprehensive studies representing other ethnic groups are required, particularly in SSA. Arguably, genomics and the use of PRS are the most promising domains in HF management. The potential benefits of this approach include better risk stratification, treatment selection and titration. However, high costs limit its use in clinical practice. Nonetheless, we propose that research into this field continues due to its immense potential for personalized medicine.

Over the years, we have seen research in HF biomarkers transition from developing biomarkers with a diagnostic utility to biomarkers with therapeutic potential. Although futuristic, we suggest that future research takes a step further by developing anti-biomarker therapies targeting established and new biomarkers involved in the pathophysiology of this complex syndrome. This strategy could potentially optimize HF treatment.

## 5. Conclusions

B-type natriuretic peptide, NT-proBNP and hs-cTn remain the gold standard diagnostic biomarkers of HF globally. Several novel biomarkers have been studied to supplement the traditional HF biomarkers for risk stratification, prognostication, and monitoring of HF therapy. However, there is scant evidence supporting the clinical utility of these new HF biomarkers. Most novel circulatory biomarkers are non-cardiac-specific, which has limited their application in clinical practice. African studies have reported the diagnostic and prognostic utility of BNP, NT-proBNP, galectin-3 and sST2 in HF. However, there is a need for sizeable primary studies assessing their role in guiding HF treatment. Most HF practice guidelines recommend using natriuretic peptides and cardiac troponins. However, adequately powered double-blinded, randomized controlled clinical trials showing definitive benefits of biomarker-guided therapy will lead to a paradigm shift in the treatment of HF. Finally, future research studies should focus on developing specific anti-biomarker pharmacotherapies that target established and new HF biomarkers.

## Figures and Tables

**Table 1:** Biomarkers recommended for heart failure (HF) by the guidelines.

Guideline (Year)	Biomarker	COR	LOE	Indication
ACC/AHA (2022) [17]	BNP/NT-proBNP	I	A	Support diagnosis or exclude HF in dyspnoeic patients
		I	A	Risk stratification of chronic HF
		I	A	Prognosis of patients admitted for HF
		IIa	B-R	Prevention of LV dysfunction or new-onset HF
		IIa	B-NR	Prognosis after discharge/measure pre-discharged levels for long-term outcomes
HFSA (2022) [17]	BNP/NT-proBNP	REC		Diagnosis of suspected HF in dyspnoeic patients
		N/REC		Routine screening in asymptomatic patients
ESC (2021) [1]	BNP/NT-proBNP	I	B	Diagnosis to rule out suspected chronic HF
		IIa		Rule out acute HF in the initial assessment of new acute HF diagnosis
	Troponin	REC		Support diagnosis or exclusion of ACS

ACC/AHA—American College of Cardiology/American Heart Association; B-NR— Level of evidence B derived from non-randomised controlled trial; B-R— Level of evidence B derived from randomised controlled trial; BNP—B-type natriuretic peptide; COR—class of recommendation; ESC—European Society of Cardiology; HF—heart failure; HFSA—Heart Failure Society of America; LOE—level of evidence; LV—left ventricular; NT-proBNP—N-terminal pro-B-type natriuretic peptide; N/REC—not recommended; REC—recommended.

**Table 2:** Summary of studies conducted in Africa investigating the clinical utility of heart failure biomarkers.

Author (Year)	Country	Sample Size	Study Population	Biomarker	Study Aim	Main Findings
Abd El-Aziz et al. (2012) [21]	Egypt	200	CAD with HFpEF (n = 100) vs. healthy control group (n = 100)	-leptin -LEP -LEPR polymorphism	Associations of serum leptin, LEP and LEPR polymorphism with HFpEF in patients with CAD	HFpEF is associated with increased serum leptin levels, and the LEP AA genotype or LEPR RR genotype carries at least a threefold increased risk of developing HFpEF. OR, 3.9, 95% CI 1.2–12.6; *p* = 0.02 for LEP AA genotype and OR, 3.7, 95% CI 1.4–10.3; *p* = 0.007 for LEPR RR genotype, respectively
Onyemelukwe et al. (2019) [22]	Nigeria	75	ADHF	BNP	Response of BNP and tissue Doppler (TD) E/e’ to standard HF therapy after 4 weeks	BNP levels decreased significantly from 450 to 275.0 pf/mL with a 38.9% reduction after 4 weeks associated with significant improvement in TD E/e’ and NYHA functional class.
Sani et al. (2020) [23]	Kenya, Mozambique, Nigeria, Senegal, South Africa, and Uganda	80	Black (73%), mixed-race (24%) & Caucasian (3%) patients with ADHF	NT-proBNP & galectin-3	Associations between NT-proBNP & galectin-3 and CV death or HF hospitalisation, NYHA, and LVEF after 6 months of HF treatment	Both biomarkers at baseline predicted combined CV death or HF hospitalisation at 6 months (hazard ratio [HR], 2.12; 95% CI 1.06–4.22; *p* = 0.032). Baseline galectin-3 & changes in NT-proBNP levels were associated with improvements in dyspnoea at 6 months.
Kingery et al. (2021) [24]	Tanzania	849	ART-naïve PLWH with diastolic dysfunction (DD) (388) & healthy adults (461)	sST2	Compare the prevalence of DD in ART-naïve PLWH to healthy adults. Association between sST2 and DD	PLWH have higher prevalence of DD (adjusted OR 2.71, 95% CI 1.62–4.55; *p* < 0.0001). Serum sST2 is associated with DD in PLWH but not uninfected subjects (*p* = 0.04 and *p* = 0.90, respectively).
Hoevelmann et al. (2021) [25]	South Africa	35	Women with acute PPCM	NT-proBNP	The role of NT-proBNP as a predictor of LVEF & LVEDD recovery in PPCM	Baseline NT-proBNP ≥ 900 pg/mL predicts failure to recover LVEDD (OR 0.22, 95% CI 0.05–0.95; *p* = 0.043) or LVEF (OR 0.20, 95% CI 0.04–0.89; *p* = 0.035) at one-year follow-up.
Bello etal. (2021) [26]	Nigeria	100	ADHF	BNP	Prognostic value of BNP	The mean BNP among non-survivors (655.0 ± 142.3 pg/mL) was higher than survivors (409.7 ± 178.2 pg/mL) *p* < 0.001. A plasma BNP level > 525 pg/mL was 87% sensitive and 75% specific for predicting death within 6-months (AUC = 0.854, 95% CI 0.756–0.951, *p* < 0.001). Kaplan-Meier survival curve showed six-month survival to be significantly reduced in patients discharged with BNP levels >525 pg/mL (57.6%) than in those with levels <525 pg/mL (98.3%), *p* < 0.001.

ADHF—acute decompensated heart failure; ART—antiretroviral; AUC—area under the curve; BNP—pro B-type natriuretic peptide; CAD—coronary artery disease; CI—confidence interval; CVD—cardiovascular; HF—heart failure; HFpEF—heart failure with preserved ejection fraction; HR—hazard ratio; LEP—leptin gene; LEPR—leptin gene receptor; LVEDD—left ventricular end-diastolic diameter; LVEF—left ventricular ejection fraction; NT-proBNP—N-terminal pro B-type natriuretic peptide; NYHA—New York Heart Association; OR— odds ratio; PLWH—people living with human immunodeficiency virus; PPCM—peripartum cardiomyopathy.

## Data Availability

Not applicable.

## Supplementary Materials

The following supporting information can be downloaded at: https://www.mdpi.com/article/10.3390/jcdd9100335/s1, Figure S1: Preferred Reporting Items for Systematic Reviews and Meta-Analyses (PRISMA) 2020 flow diagram.
