# Supplementary material for: New Biomarkers and Their Potential Role in Heart Failure Treatment Optimisation—An African Perspective
Source: J Cardiovasc Dev Dis. 2022 Oct 2;9(10):335. doi: 10.3390/jcdd9100335 (PMC9604249; doi:10.3390/jcdd9100335)
Supplement: Supplementary file 1 [file jcdd-09-00335-s001.zip › jcdd-1871758-supplementary.pdf]

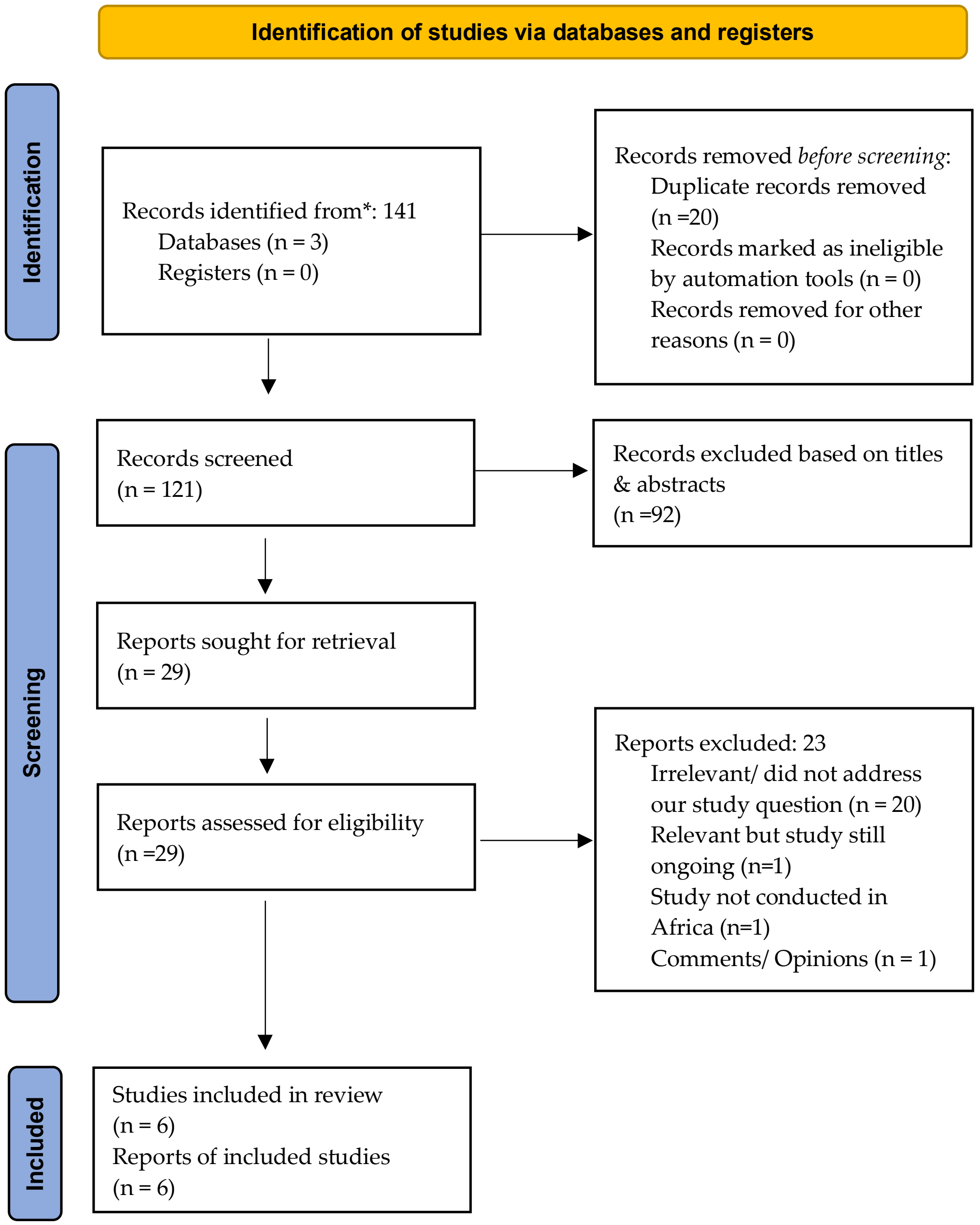

Figure S1: Preferred Reporting Items for Systematic Reviews and Meta-Analyses (PRISMA) 2020 flow diagram.
